# Fatty Acid and Aroma Profiles of Microencapsulated Olive Oils from Southeastern Anatolia: Effects of Cultivar Variations, Storage Time, and Wall Material Formulation

**DOI:** 10.3390/foods14142439

**Published:** 2025-07-10

**Authors:** Songül Kesen, Eda Elgin Kiliç

**Affiliations:** Naci Topcuoglu Vocational School, Gaziantep University, 27600 Gaziantep, Turkey

**Keywords:** Nizip Yaglik, Kilis Yaglik, microencapsulation, fatty acid, volatile compounds, SPME

## Abstract

The microencapsulation of olive oil plays an important role in food science and technology by controlling oxidative deterioration, improving emulsification, and preserving bioactive properties, ultimately benefiting product formulations in both the culinary and medical fields. This study is important in that it reveals the effect of the microencapsulation process on aroma compounds and provides a data set for investigating the potential use of powdered products. In this study, the microencapsulation of emulsions prepared with carbohydrate–protein-based coating materials of oils obtained from two different olive varieties (Nizip and Kilis Yaglik) grown in the Southeastern Anatolia Region of Turkey was carried out via the freeze-drying method. In the study, emulsions were formed using protein isolate (WPI) and maltodextrin (MD) at different ratios (1:1, 1:4, 1:10) as wall materials, and microcapsule powder products were obtained via the freeze-drying method. While the physical properties of the emulsions and microcapsules were examined, the oxidative stability, fatty acid profile, and aroma compounds were examined in oils and microcapsules. The changes in oxidative stability and aroma compounds were also monitored during storage (0, 45, and 90 days at room temperature). According to the data obtained, it was observed that the emulsion stability increased with increasing maltodextrin content. Similarly, the microencapsulation efficiency was also found to change in direct proportion to the maltodextrin ratio. Encapsulated samples showed better oxidative stability than oils. Oleic acid was the predominant fatty acid in both oils and microencapsulated products, followed by palmitic and linoleic acids. According to the aroma compounds, the microcapsules obtained from both types of oils were clearly separated from the oils.

## 1. Introduction

Microencapsulation is defined as the process of surrounding a core component with one or more coating materials. This technique aims to protect, store, and release components with various biological, chemical, or physical properties in a controlled manner. Microencapsulation is used in numerous fields such as the food industry; the main reasons for this use are increasing the stability of ingredients, extending the shelf life of products, and preserving their contents [1,2].

In the microencapsulation process, the drying method is one of the most important steps. Drying methods are critical factors that affect the quality, stability, and shelf life of microencapsulated products in general [3,4,5]. The main techniques commonly used in the microencapsulation process are spray drying, freeze-drying, coacervation, fluidized bed coating, and supercritical fluid technology.

Freeze-drying, or lyophilization, is a widely used technique for microencapsulation, especially for heat-sensitive and volatile compounds. One of the most significant advantages of freeze-drying is that it operates at low temperatures. This minimizes the thermal degradation of sensitive bioactive compounds, ensuring that their structural integrity and functionality are maintained during the drying process. Many sensitive ingredients, such as probiotics and essential oils, are better preserved when freeze-dried [6,7].

Microencapsulation is a process used in olive oil for various purposes, especially to improve the quality of the product, expand its uses, and protect its health benefits. The main reasons for microencapsulation in olive oil include protection against microorganisms, the prevention of oxidation, and the stability of nutrients [8].

Microencapsulation is also used to extend the shelf life of olive oil. Oxidation is a process that negatively affects the quality and nutritional profile of olive oil. Since the active ingredients in olive oil are sensitive to external environmental factors, microencapsulation helps consumers to take food safety measures by preventing the degradation of nutrients in olive oil [9]. The aroma and flavor of olive oil can be preserved for longer periods of time thanks to microencapsulation, which allows food businesses in particular to improve their product quality.

The wall material has a direct impact on the microencapsulation efficiency. Several studies show that the proper choice of wall material improves the performance of the microencapsulation process [10]. The wall material protects the active ingredients against environmental influences, shielding them from damage caused by oxidation, moisture, and light. Especially for essential oils or bioactive ingredients that tend to degrade easily, the choice of an effective wall material is essential [11,12]. The choice of wall material affects the dissolution rate and release dynamics of the active ingredients. For example, some wall materials allow for the production of microcapsules that are water-sensitive and have slow-release properties [13]. Maltodextrin and whey protein isolate are widely used in the food industry as preferred wall materials in microencapsulation processes. The combined use of these materials not only increases the microencapsulation efficiency, but also provides significant benefits in protecting active compounds [14].

Microencapsulated components have been shown to be released in a more controlled manner under certain conditions [15]. Microencapsulation can help determine the rate at which aroma components are released under targeted conditions. Parameters such as surface properties and capsule wall thickness can influence the diffusion of aroma components, controlling how they are released over a given time period [16]. This is especially important in applications such as aromatherapy and the food industry to ensure a fresh and long-lasting aroma. In previous studies, the microencapsulation of orange essential oil using modified rice starch helped to preserve the components and better retain the aroma characteristics [17]. In a study on propolis microencapsulation, it was emphasized that positive effects were achieved in terms of both taste and aroma, and that in this respect, it could have important potential for use in the food and beverage sector [18]. In another study on the microencapsulation of spices, it was observed that the ability of volatile components to remain stable increased [19].

In this paper, we have studied the effects of different olive oils (Nizip Yaglik and Kilis Yaglik) in the microencapsulation process via freeze drying for the first time. The aroma profiles for the different olive oils before and after the microencapsulation process have been assessed in order to evaluate the olive oils’ reactions to the freeze-drying process. The aim of the study is to (i) perform the microencapsulation of the Nizip and Kilis oils for the first time, (ii) to examine the fatty acid composition, aroma compounds, and oxidative stability of the oils and microcapsules, and (iii) to determine the changes in the aroma profiles and oxidative stability during storage.

## 2. Materials and Methods

### 2.1. Material

The Nizip Yaglik (NY) and Kilis Yaglik (KY) varieties used in the study were obtained from the facilities in the growing regions where the oil was obtained via the two-phase continuous oil extraction system (Oliomio mini, Florence, Italy). Whey protein isolate (WPI, protein ocean) and maltodextrin (MD, alfasol) as wall materials and other chemicals were obtained from companies selling chemical materials.

### 2.2. Method

#### 2.2.1. Emulsion Preparation

Olive oil microencapsulation was realized by the core material (olive oil) forming a thin and stable emulsion in the wall solution. To adjust the protein/polysaccharide ratio of the microcapsule wall, whey protein isolate (WPI) and maltodextrin (MD) were used to prepare six samples, as shown in Table 1 for both types of olive oil [20,21]. The prepared wall material was homogenized in a mechanical homogenizer (Ultra Turrax T18, IKA, Istanbul, Turkey ) at 10,000 rpm for 5 min. A mixture of oil and Tween 80 was added dropwise to the wall materials during homogenization at 20,000 rpm for 10 min to ensure successful homogenization.

##### Emulsion Stability

The emulsion stability was determined according to the method described by Alcântara et al. [22]. The prepared emulsions were placed in a 25 mL measuring cylinder, sealed with plastic wrap, and left at room temperature for one day. After 24 h, the height of the separated upper phase in the measuring cylinder was read and recorded. The emulsion stability was calculated as “% separation” according to the following equation.

%Separation = (H_1_/H_0_) ∗ 100

H_0_: Initial height of the emulsion; H_1_: Height of the upper phase measured after 24 h.

#### 2.2.2. Freeze-Drying (Lyophilization)

The emulsion was frozen in Falcon tubes at −80 °C for 12 h before being connected to the freeze-drying unit. Drying was carried out for 48 h using a freeze-dryer (Labconco, Kansas City, MO, USA) under a vacuum of 0.1 mbar and a condenser set at −52 °C. The microparticles produced via freeze-drying were kept in a refrigerator at 4 °C.

#### 2.2.3. Microencapsulation Efficiency

The microencapsulation efficiency was calculated using the ratio of oil excluding the surface oil to the total oil in the powder product obtained from the microencapsulation process. To determine the amount of surface oil, 1 g of the powder product was put into a beaker, mixed in 5 mL petroleum ether, and filtered, and the residue obtained was mixed with petroleum ether again, filtered, and kept in an oven at 70 °C for 30 min to evaporate the petroleum ether until it reached a constant weight. Finally, the Petri dish was weighed and the amount of surface oil was calculated. The total amount of oil in the equation is assumed to be equal to the oil added according to the initial amount of carrier [23].

ME = (TOA − SOA)/TOA

ME: Microencapsulation Efficiency, %; TOA: Total Oil Amount, g; SOA: Surface Oil Amount, g.

#### 2.2.4. Oxidative Stability

The oxidative stability of encapsulated oils was determined via peroxide analysis. For the analysis, 2 g of oil was dissolved in 30 mL acetic acid/chloroform (3:2 *v*/*v*). Then, 0.5 mL of saturated potassium iodide solution was added. After stirring for 1 min, the mixture was kept at room temperature and in the dark for 5 min, and 30 mL of water was added. As an indicator, 2 mL starch solution (1 g/100 mL) was added and titrated with 0.1 N standardized sodium thiosulfate solution until the color disappeared [24].

Peroxide Value = V ∗ N ∗ 1000/Sample amount

V: amount of sodium thiosulfate spent (ml). N: Normality of sodium thiosulfate (0.1 N).

#### 2.2.5. Analysis of Fatty Acids

For the analyses, the oils and oils from the microcapsules were converted to fatty acid methyl esters and then analyzed using gas chromatography (GC). The fatty acid methyl esters were isolated using the cold transesterification method. To prepare the fatty acid methyl esters, approximately 0.1 g of the oil sample was weighed in a 5 mL glass tube and mixed with 2 mL of n-hexane. Then, 0.2 mL of 2 N methanolic KOH was added to the tubes, sealed tightly, and stirred rapidly for 30 s. The tubes were then centrifuged and allowed to stand for clear phase separation, and 1 μL of the methyl esters collected in the supernatant was taken with a Pasteur pipette and injected into the gas chromatograph [25]. Peaks were identified using standard compounds (FAME mix), and the amount of fatty acids was calculated as percentage (%).

#### 2.2.6. Analysis of Aroma Compounds

The solid phase microextraction (SPME) method was used for the extraction of the aroma compounds of oil and microcapsules. Polydimethylsiloxane/divinylbenzene/carboxene (PDMS/DVB/CAR) fiber with a phase thickness of 50/30 μm was used in the extraction process. First, 3 g of the samples was placed into 10 mL bottles, sealed, and the SPME fiber was exposed to the upper cavity of the bottle for 30 min. The temperature of the samples was set to 40 °C. The fiber-adsorbed volatile compounds were injected into the gas chromatography (GC) injection port [26].

##### GC-FID and GC-MS Conditions

The quantification and identification of the flavoring substances were carried out via “Agilent 6890N” brand gas chromatography and an “Agilent 5975B VL MSD” mass spectrometer (MS).

An Agilent 6890N gas chromatograph with FID was used for the quantification of the flavoring substances. The separation of flavorings was performed using a DB-WAX capillary column (60 m × 0.25 mm × 0.4 µm). The injector temperature was 220 °C, the detector temperature was 250 °C, and the column temperature was programmed to increase by 2 °C per minute to 220 °C, and then by 3 °C per minute to 245 °C after 3 min at 60 °C, and to remain constant at this temperature for 20 min. The amount to be injected into the device was 3 µL. Helium with a flow rate of 1.5 mL/min was used as the carrier gas.

The “Agilent 5975B VL MSD” brand mass spectrometer connected to gas chromatography was used for the identification of the flavoring substances. The velocity of helium used as carrier gas was 1.5 mL/min. The ionization energy of the mass spectrometer was kept at 70 eV, the ion source temperature was 250 °C, the quadrupole temperature was 120 °C, and scans between 29 and 350 mass/charge (m/e) were performed at 1 s intervals. Peaks were identified by injecting the standard solution for compounds with the standard and comparing the mass spectra of compounds without the standard with the mass spectra of flavor. The Flavor 2L, Wiley 7.0, and NIST-98 flavoring libraries in the computer memory were used. The amounts of flavorings were calculated via the internal standard method using the following equation [27]. Analyses followed during storage.

#### 2.2.7. Statistical Analysis

The SPSS 22 package program was used to evaluate the results, and significant differences were evaluated according to the Duncan multiple comparison test. On the other hand, the differences and/or similarities of the data obtained during storage were classified and evaluated via principal component analysis (PCA), one of the multivariate data analysis methods [28].

## 3. Results and Discussion

### 3.1. Results of Emulsion Stability

The percentage of separation in the emulsions produced with different ratios of wall materials is shown in Table 2. The emulsion stability is related to the physical stability of emulsion systems, which allows the emulsion to remain homogeneous over long periods of time. A high percentage of separation of emulsions indicates that the system is unstable, while a low percentage of separation indicates high stability.

In emulsion formulations with both oil types, it was observed that the percentage of separation decreased as the MD ratio increased (from 1:1 to 1:10). This indicates that the stability of the emulsion increases with the addition of more MD. Similarly, Chaabane et al. [20] used coating agents such as maltodextrin and whey protein isolate in the microencapsulation of olive oil and investigated the effects of different ratios on the emulsion stability. In the study, it was stated that the stability of emulsions varied depending on the proportions of the ingredients in the formulation.

### 3.2. Determination of Microencapsulation Efficiency (ME)

The change in the microencapsulation efficiency of microencapsulated olive oil powders is shown in Table 3. Increasing the MD ratio in the wall material increased the microencapsulation efficiency, resulting in more stable capsules. The ME value, varying according to the wall composition, is in the range of 87.09–90.56 for Nizip oil powders and 87.30–90.59 for Kilis oil powders. The highest microencapsulation efficiency was determined to be at a WPI:MD ratio of 1:10 in both olive oil powders. As in our study, the increase in the ratio of maltodextrin to wall material improved the protective properties of the wall materials, as stated by Calvo [29]. As the proportion of maltodextrin in the emulsion formulation increases, the microencapsulation efficiency value is statistically significant (*p* < 0.05). On the other hand, Chaabane et al. [20] investigated different emulsification ratios prior to the freeze-drying of olive oil, and showed that a mixture of 50% MD and 50% WPI provided an optimal balance between structural integrity and encapsulation efficiency, achieving an approximately 82% encapsulation efficiency in microcapsules.

### 3.3. Results of Oxidative Stability

The oxidative stability of oil and microencapsulated powder products is expressed in peroxide values (Table 4). The evaluation of peroxide values (PVs), expressed in meq O_2_/kg oil, is crucial, as it is a primary indicator of lipid oxidation and therefore a measure of oil quality. This study presents the peroxide values of two different oils (NY and KY) under varying ratios of microencapsulation (1:1, 1:4, 1:10) over time intervals of 0, 45, and 90 days. According to the results, an increase in the peroxide values was observed in both oils and microencapsulated samples during storage. Encapsulated samples showed better oxidative stability than oil. In encapsulated oils, the best oxidative stability was observed in the NY (1:10) and KY (1:10) formulation ratios. The initial peroxide values for both NY and KY microcapsules show relatively low values, ranging from 5.94 to 6.55 meq O_2_/kg oil, regardless of the formulation ratios. This indicates that on day 0, the oils are in a steady state; with minimal pre-existing oxidation on day 0, the peroxide values in all samples measured were not statistically significant. By day 45, the peroxide values increase for all formulations, indicating the onset of oxidation. The most pronounced increases are observed by day 90, with values ranging from 8.39 to 16.06 meq O_2_/kg. The best protection was observed at 1:10 formulation ratios, with the lowest value of 8.39 meq O_2_/kg oil peroxide in NY (1:10), indicating that 1:10 is the best formulation ratio to maintain oxidative stability. The results obtained are consistent with the microencapsulation efficiency. The formulation ratio with a high microencapsulation efficiency has a low peroxide value. Similarly, Hee et al. [30] reported that the peroxide values decreased as the microencapsulation efficiency of coconut oil increased.

### 3.4. Fatty Acid Profiles of Oils and Microencapsules

As a result of fatty acid analysis, saturated and unsaturated fatty acids were determined (Table 5 and Table 6). According to these results, unsaturated fatty acids (MUFAs + PUFAs) were higher than saturated fatty acids (SFAs) for both NY and KY oils. The unsaturated fatty acid and SFA values for NY were 73.35% and 26.64%, while the values for KY were 72.62% and 27.37%, respectively. Among unsaturated fatty acids, oleic acid was found to be the dominant fatty acid in both NY and KY oils and the microencapsulated powder products obtained from these oils. This was followed by palmitic and linoleic fatty acids, respectively (Table 5 and Table 6). Oleic acid (C18:1) is an important compound in this group and is recorded as having the highest proportion in both oils.

The results obtained for fatty acids are consistent within the official limits for extra virgin olive oil (oleic acid, 55.0–83.0; palmitic acid 7.5–20.0%; linoleic acid 2.5–21.0%, and palmitoleic acid 0.3–3.5%) [29]. A previous study stated that major fatty acids in olive oils are oleic (55–85%), palmitic (7.5–20%), linoleic (7.5–20%), stearic (0.5–5%), palmitoleic (0.3–3.5%), and linolenic (0.0–1.5%) acids, although traces of myristic, arachidic, and margaric acids have also been found [31]. These values are consistent with the data obtained in the present study.

After the freeze-drying of both oil samples and emulsions with different formulations, the amount of oleic acid (for NY 57.60–58.51%; for KY 57.82–58.64), the most dominant compound, was preserved, and no statistically significant change was observed (*p* > 0.05). Similarly, the concentration of lauric, palmitoleic, stearic, and arachidic acids in NY oil and capsules, and only lauric acid in KY oil and capsules, were statistically similar. As a result, when the general profile of fatty acids after microencapsulation was examined, it was found that the concentrations of 14 fatty acids in different formulations were slightly affected by the microencapsulation process. Microencapsulation reduces the oxidation of oils and fatty acids and their exposure to environmental influences. This is particularly important for oxidation-sensitive components such as polyunsaturated fatty acids (PUFAs) [32]. Similar results were obtained by Keshri et al. [33] in flaxseed oil and Chasquibol et al. [34] in sacha inchi oil.

### 3.5. Aroma Compounds of the Oils and Microencapsules

The data presented in the aroma compounds (Table 7 for NY and Table 8 for KY) gives the concentrations of various volatile compounds measured in parts per billion (µg/kg, ppb) for different formulations of wall material and during storage. Aroma compounds play a crucial role in determining the sensory characteristics of food products. Understanding these compounds can improve flavor profiles and increase consumer acceptance. The compounds listed in the tables are mainly aldehydes, alcohols, terpenes, and acids, which can significantly affect the perception of aroma. Table 7 and Table 8 highlight the potential changes in the concentrations of NY and KY aroma compounds at specific intervals (0, 45, and 90 days) under three different wall material formulations (NY (1:1), NY (1:4), and NY (1:10)). The data show significant changes in the concentrations of these compounds over time. The increase in some compounds may indicate that they are the result of oxidative processes or enzymatic reactions. The decrease or non-detectability of some compounds in certain groups suggest that the compound was degraded, not produced, or lost during the microencapsulation process or during storage.

According to Table 7 and Table 8, the highest amount of hexanal and hexanol compounds were detected in NY and KY oils, respectively. As seen in Table 7, in the hexanal compound, the initial values in the NY (1:1), NY (1:4), and NY (1:10) groups were quite different from the values in the NY group; for example, while 1481 µg/kg was obtained on day 0 in NY (1:1), 2812 µg/kg was obtained in NY (1:10). A similar change was observed in KY oil. This indicates that different mechanisms operate in the formation, loss, or transformation of aroma compounds during the microencapsulation process. Although the hexanal amount decreased with the effect of the microencapsulation process and storage time, the highest amounts were observed in the NY (1:10) and KY (1:10) formulations. Microencapsulation has been demonstrated to alter the volatile profile of olive oil, including the amount of hexanal present. Research shows that microencapsulated formulations can lead to a reduced concentration of hexanal due to improved oxidative stability [20]. At the end of the storage period (90 days), hexanal amounts were analyzed, and the lowest hexanal amount was determined in NY (1:1) and KY (1:1) formulations as 1227 µg/kg and 1154 µg/kg, respectively.

The amount of D-Limonene for NY and KY showed remarkable stability, although it varied during storage and at different formulation ratios. The highest retention rate was observed at the end of the 90th day and was 93.16% and 77.06% for the NY1:1 and KY1:1 formulations, respectively. Previous studies have similarly shown that the encapsulation of D-limonene can significantly reduce its degradation and loss during processing and storage [35,36]. Tang et al. [35] showed that complex coacervation can retain about 73.7% to 88% of D-limonene during the spray drying process.

*Ɣ*-Terpinene and *p*-cymene were not observed in the oil samples, but were reported at significant levels in the microencapsulated powders of NY and KY, and increased over time. The Ɣ-terpinene values measured at day 90 in the NY (1:10) and KY (1:10) formulations were 5297 µg/kg and 5098 µg/kg, respectively, while for *p*-cymene, these values were 3270 µg/kg and 3078 µg/kg, respectively. This increase indicates that formation mechanisms specific to this compound are activated in the microencapsulation treatment. The microencapsulation process has been reported to protect volatile compounds such as γ-terpinene against degradation through oxidation and evaporation, and thus stabilize the flavor profile of the oil during storage [37]. One of the most critical functions of γ-terpinene is its role as an antioxidant. According to Mollica et al. [38], γ-terpinene can enhance the antioxidant effect of α-tocopherol (vitamin E) in edible oils. One of the most notable features of *p*-cymene is its antioxidant potential. Wang et al. [39] demonstrated that *p*-cymene can significantly reduce oxidative stress, thus lowering the occurrence of colorectal cancer. Although the exact amounts of γ-terpinene in microencapsulated olive oil appear limited in the available literature, the general findings suggest that microencapsulated formulations can significantly increase the retention rates of such volatile compounds [40,41].

Alcohol compounds, such as hexanol and heptanol, were detected in the oil samples with relatively high concentrations, and changes over time appear moderate in some cases, with statistically significant differences (*p* < 0.5). Hexanol in NY increased slightly from 5266 µg/kg to 6265 µg/kg, whereas heptanol increased from 1312 µg/kg to 2221 µg/kg. In KY, hexanol varied between 5536 µg/kg and 4976 µg/kg, whereas heptanol increased from 1476 µg/kg to 1891 µg/kg.

Acid compounds were detected only in oils, and not in microencapsulated samples. In a previous study, it was reported that organic acids such as acetic acid are secondary oxidation products, and give sour or pungent odors in the olive oil flavor profile [42]. Similarly, compounds such as nonanoic acid and hexanoic acid exhibited different profiles during the storage process of NY and KY oils, suggesting that the chemical or enzymatic pathways leading to their formation are sensitive to the sample matrix. The absence of these compounds in microencapsulated samples suggests that the microencapsulation process protects olive oil against oxidation.

Compounds such as (E)-2-heptenal and nonanal are commonly formed during the oxidation of olive oil lipids. These aldehydes are produced through the breakdown of fatty acid hydroperoxides derived from unsaturated fatty acids such as linoleic and linolenic acids [43]. In our study, (E)-2-heptenal was detected only in NY and not in microencapsulated samples. Nonanal increased with storage time in both oil samples, but the rate of increase was lower in microencapsulated samples. Heptanal was measured only in the oil samples; it was not detected in the microencapsulated groups. This indicates the protective effect of microencapsulation on oxidation.

Other compounds such as 2-pentyl furan, (Z)-3-hexenol, and (E)-2-decenal were not detected in the microencapsulated samples, indicating the influence of the processing conditions on the flavor profile.

### 3.6. PCA Results of the Aroma Compounds and Fatty Acids

**Figure 1:**
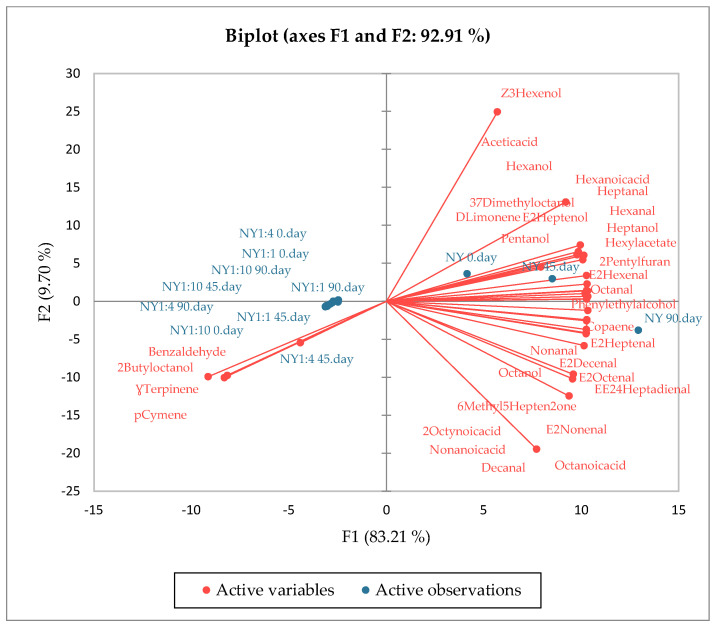
The scores and factor plane plots for aroma compounds of oil and microencapsules of NY.

**Figure 2:**
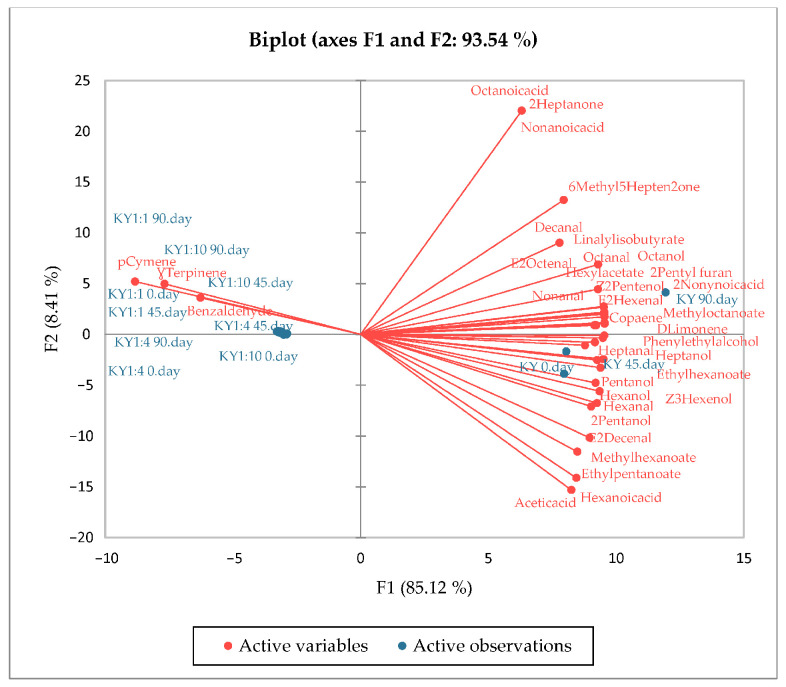
The scores and factor plane plots for aroma compounds of oil and microencapsules of KY.

According to the PCA results of aroma compounds (Figure 1), both oil samples and encapsulated samples were in distinctly different groups during storage. For PCA, 33 variables were used for the NY oil (Figure 1) and 35 variables for the KY oil (Figure 2), and the two principal components explained 92.91% (F1, 83.21%; F2, 9.70%) and 93.54% (F1, 85.12%; F2, 8.41%) of the total variance for NY and KY, respectively. This situation demonstrates that PCA successfully reveals the differences between volatile compound profiles. Microcapsules obtained from the NY variety were characterized by *p*-cymene, 2-butyl octanol, Ɣ-terpinene, and benzaldehyde compounds, while microcapsules obtained from the KY variety were characterized by *p*-cymene, Ɣ-terpinene, and benzaldehyde compounds. The microcapsules obtained from both oil varieties were clearly separated from the oils. In both oil samples, the samples at 90 days were in different groups from the samples at 0 and 45 days.

**Figure 3:**
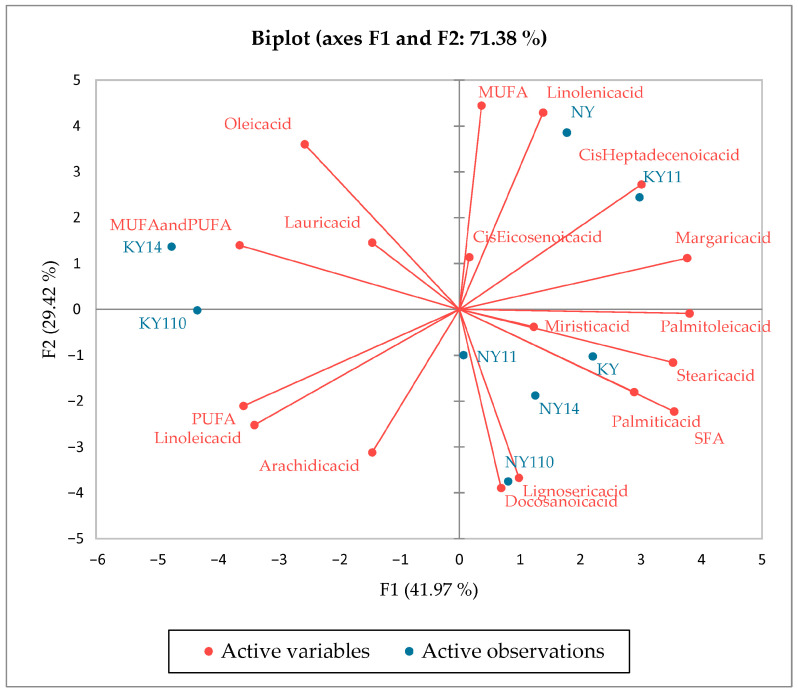
The scores and factor plane plots for fatty acids of oil and microencapsules of NY and KY.

The 18 variables of fatty acids were selected for the PCA, and the explained variance was 71.38% (F1: 41.97%; F2: 29.42%). The application of the PCA algorithm showed four distinct groups (Figure 3). The microcapsules are significantly different from the oils from which they are obtained in terms of their fatty acid profiles. KY (1:4) is characterized by MUFAs and PUFAs, while NY (1:10) is characterized by lignoseric and docosanoic acid, KY (1:1) by Cis-10-heptadecenoik acid, and NY by linoleic acid and MUFAs.

## 4. Conclusions

In this study, the best emulsion stability and microencapsulation efficiency were found in the NY (1:10) and KY (1:10) formulations. On the other hand, an increase in peroxide values was observed in both the oils and the microencapsulated samples during storage. The encapsulated samples showed better oxidative stability than the oils. The best oxidative stability in the encapsulated oils was observed in the NY (1:10) and KY (1:10) formulation ratios. Oleic acid was found to be the dominant fatty acid in both oils and microencapsulated powder products. The findings of the aroma analysis emphasize that various microencapsulation formulations (1:1, 1:4, and 1:10) affect the stability and concentration of key aroma components over time. In particular, compounds such as hexanal, D-limonene, γ-terpinene, and *p*-cymene exhibited significant concentration changes both during storage and across different wall material formulations. The initial presence of significant amounts of hexanal indicates that it plays an important role in the initial flavor profile of both NY and KY oils. However, its concentration decreased during storage, indicating oxidative degradation, contributing to the loss of certain aroma compounds. Conversely, microencapsulation enhanced the stability of D-limonene, with high retention rates observed even after 90 days. The significant presence of compounds such as γ-terpinene and *p*-cymene in microencapsulated powders suggests the existence of a mechanism activated during the microencapsulation process that facilitates their formation. Future research should focus on further elucidating the mechanical pathways underlying the retention and degradation of aroma compounds during microencapsulation and storage, and on discovering new wall materials that can enhance protective effects without compromising sensory qualities. By developing these techniques, the olive oil industry has the potential to significantly improve product quality, sustainability, and consumer satisfaction.

## Figures and Tables

**Table 1:** Emulsions prepared in different combinations of different wall materials.

Olive Oil Samples	Wall Materials	Wall Materials Ratio	Amount of Wall Material (g)	Amount of Olive Oil (g)	Amount of Water (g)
NY	WPI	1:1	12.5	12.5	62.5
	Maltodextrin		12.5		
KY	WPI	1:1	12.5	12.5	62.5
	Maltodextrin		12.5		
NY	WPI	1:4	5	12.5	62.5
	Maltodextrin		20		
KY	WPI	1:4	5	12.5	62.5
	Maltodextrin		20		
NY	WPI	1:10	2.27	12.5	62.5
	Maltodextrin		22.73		
KY	WPI	1:10	2.27	12.5	62.5
	Maltodextrin		22.73		

**Table 2:** Separation values of emulsions after 24 h (%).

Emulsion Formulations	Separation, % *
NY (1:1)	11.55 ± 0.06 ^e^
NY (1:4)	11.44 ± 0.03 ^d^
NY (1:10)	10.98 ± 0.04 ^b^
KY (1:1)	11.44 ± 0.03 ^d^
KY (1:4)	11.09 ± 0.04 ^c^
KY (1:10)	10.45 ± 0.06 ^a^

* Results are the mean value of three replications and standard deviation (mean ± std dev.) of emulsion separation values after 24 h. Emulsion separations with different lowercase letters (a–e) in the same column are statistically significant (*p* < 0.05).

**Table 3:** Microencapsulation efficiency of microcapsules (%).

Microencapsules	Microencapsulation Efficiency (ME, %) *
NY (1:1)	87.09 ± 1.17 ^a^
NY (1:4)	89.46 ± 1.62 ^ab^
NY (1:10)	90.56 ± 0.16 ^b^
KY (1:1)	87.30 ± 1.99 ^a^
KY (1:4)	89.33 ± 1.29 ^ab^
KY (1:10)	90.59 ± 0.20 ^b^

* Results are the mean value of three replications and standard deviation (mean ± std dev.) of microencapsulation efficiency. Microencapsulation efficiencies of microcapsules with different lowercase letters (a, b) in the same column are statistically significant (*p* < 0.05).

**Table 4:** Peroxide values of olive oils and microcapsule products.

Microcapsules	Peroxide Values (meq O_2_/kg oil) *		
	Days		
	0	45	90
NY	5.94 ± 0.36 ^aA^	9.25 ± 0.35 ^cB^	15.98 ± 0.75 ^dC^
NY (1:1)	6.35 ± 0.10 ^aA^	7.94 ± 0.55 ^abA^	10.96 ± 1.10 ^cB^
NY (1:4)	6.26 ± 0.31 ^aA^	7.51 ± 0.05 ^abB^	9.81 ± 0.00 ^bcC^
NY (1:10)	6.06 ± 0.43 ^aA^	7.04 ± 0.40 ^aAB^	8.39 ± 0.46 ^aB^
KY	6.04 ± 0.15 ^aA^	9.19 ± 0.44 ^cB^	16.06 ± 0.09 ^dC^
KY (1:1)	6.55 ± 0.16 ^aA^	8.01 ± 0.51 ^bB^	10.82 ± 0.55 ^cC^
KY (1:4)	6.53 ± 0.15 ^aA^	7.60 ± 0.38 ^abB^	9.12 ± 0.28 ^abC^
KY (1:10)	6.18 ± 0.56 ^aA^	7.18 ± 0.15 ^abA^	8.61 ± 0.17 ^abB^

* Results are the mean value of two replications and standard deviation (mean ± std dev.) of peroxide values. Peroxide values of oil and microcapsule samples with different lowercase letters (a–d) in the same column are statistically significant (*p* < 0.05). Peroxide values of oil and microcapsule samples with different capital letters (A–C) in the same row are statistically significant (*p* < 0.05).

**Table 5:** Fatty acid composition of NY olive oil and microcapsule products.

	Fatty Acids	Concentration of Fatty Acids (%) *			
		NY	NY (1:1)	NY (1:4)	NY (1:10)
1	Lauric acid (C12:0)	0.01 ± 0.00 ^a^	0.01 ± 0.00 ^a^	0.01 ± 0.00 ^a^	0.01 ± 0.00 ^a^
2	Miristic acid (C14:0)	0.02 ± 0.00 ^a^	0.05 ± 0.01 ^b^	0.11 ± 0.01 ^d^	0.06 ± 0.00 ^c^
3	Palmitic acid (C16:0)	16.43 ± 0.26 ^a^	16.54 ± 0.29 ^ab^	16.82 ± 0.07 ^b^	16.76 ± 0.04 ^b^
4	Palmitoleic acid (C16:1)	2.41 ± 0.19 ^a^	2.17 ± 0.24 ^a^	2.27 ± 0.03 ^a^	2.40 ± 0.02 ^a^
5	Margaric acid (C17:0)	0.41 ± 0.06 ^b^	0.36 ± 0.01 ^ab^	0.35 ± 0.00 ^a^	0.36 ± 0.01 ^ab^
6	Cis-10-Heptadecenoik acid (C17:1)	0.58 ± 0.02 ^c^	0.49 ± 0.05 ^b^	0.49 ± 0.02 ^b^	0.40 ± 0.02 ^a^
7	Stearic acid (C16:0)	7.83 ± 0.12 ^a^	7.88 ± 0.64 ^a^	7.84 ± 0.01 ^a^	7.88 ± 0.05 ^a^
8	Oleic acid (C18:1)	58.51 ± 2.13 ^a^	57.91 ± 0.84 ^a^	57.60 ± 0.34 ^a^	57.67 ± 0.41 ^a^
9	Linoleic acid (C18:2)	9.73 ± 0.30 ^a^	10.72 ± 0.18 ^b^	10.66 ± 0.31 ^b^	10.91 ± 0.08 ^b^
10	Arachidic acid (C20:0)	1.37 ± 0.03 ^a^	1.43 ± 0.05 ^a^	1.43 ± 0.02 ^a^	1.44 ± 0.04 ^a^
11	Cis-11-Eicosenoic acid (C20:1)	0.73 ± 0.01 ^c^	0.66 ± 0.02 ^b^	0.66 ± 0.01 ^b^	0.62 ± 0.01 ^a^
12	Linolenic acid (C18:3)	1.39 ± 0.03 ^b^	1.18 ± 0.18 ^a^	1.17 ± 0.02 ^a^	1.16 ± 0.08 ^a^
13	Docosanoic acid (C22:0)	0.38 ± 0.02 ^a^	0.40 ± 0.03 ^a^	0.39 ± 0.03 ^a^	0.46 ± 0.01 ^b^
14	Lignoseric acid (C24:0)	0.20 ± 0.01 ^a^	0.21 ± 0.03 ^a^	0.21 ± 0.01 ^a^	0.29 ± 0.04 ^b^
	Saturated fatty acids (SFAs)	26.64	26.88	27.16	27.26
	Monounsaturated fatty acids (MUFAs)	62.23	61.23	61.02	61.09
	Polyunsaturated fatty acids (PUFAs)	11.12	11.90	11.83	12.07
	Unsaturated fatty acids (MUFAs + PUFAs)	73.35	73.13	72.85	73.16

* Results are the mean value of three replications and standard deviation (mean ± std dev.) of fatty acids. Concentration of oils and microcapsule samples with different lowercase letters (a–c) in the same row are statistically significant (*p* < 0.05).

**Table 6:** Fatty acid composition of KY olive oil and microcapsule products.

	Fatty Acids	Concentration of Fatty Acids (%) *			
		KY	KY (1:1)	KY (1:4)	KY (1:10)
1	Lauric acid (C12:0)	0.02 ± 0.01 ^a^	0.02 ± 0.01 ^a^	0.02 ± 0.01 ^a^	0.02 ± 0.01 ^a^
2	Miristic acid (C14:0)	0.00 ± 0.00 ^a^	0.12 ± 0.01 ^d^	0.04 ± 0.00 ^c^	0.03 ± 0.01 ^b^
3	Palmitic acid (C16:0)	16.55 ± 0.03 ^ab^	16.83 ± 0.09 ^b^	16.37 ± 0.43 ^ab^	16.32 ± 0.19 ^a^
4	Palmitoleic acid (C16:1)	2.22 ± 0.01 ^b^	2.41 ± 0.07 ^c^	1.81 ± 0.13 ^a^	1.84 ± 0.09 ^a^
5	Margaric acid (C17:0)	0.41 ± 0.02 ^b^	0.40 ± 0.02 ^b^	0.30 ± 0.01 ^a^	0.30 ± 0.02 ^a^
6	Cis-10-Heptadecenoik acid (C17:1)	0.54 ± 0.02 ^c^	0.58 ± 0.01 ^d^	0.45 ± 0.02 ^b^	0.35 ± 0.01 ^a^
7	Stearic acid (C16:0)	8.25 ± 0.14 ^b^	7.93 ± 0.24 ^b^	7.33 ± 0.28 ^a^	7.55 ± 0.27 ^ab^
8	Oleic acid (C18:1)	57.82 ± 1.40 ^a^	58.05 ± 1.22 ^a^	58.64 ± 2.18 ^a^	58.49 ± 1.54 ^a^
9	Linoleic acid (C18:2)	10.15 ± 0.30 ^a^	9.90 ± 0.41 ^a^	11.24 ± 0.25 ^b^	11.18 ± 0.28 ^b^
10	Arachidic acid (C20:0)	1.50 ± 0.05 ^b^	1.32 ± 0.03 ^a^	1.43 ± 0.03 ^b^	1.46 ± 0.09 ^b^
11	Cis-11-Eicosenoic acid (C20:1)	0.68 ± 0.02 ^b^	0.58 ± 0.04 ^a^	0.64 ± 0.03 ^b^	0.65 ± 0.03 ^b^
12	Linolenic acid (C18:3)	1.21 ± 0.08 ^a^	1.35 ± 0.04 ^b^	1.20 ± 0.03 ^a^	1.23 ± 0.02 ^a^
13	Docosanoic acid (C22:0)	0.42 ± 0.02 ^b^	0.36 ± 0.02 ^a^	0.36 ± 0.01 ^a^	0.40 ± 0.01 ^b^
14	Lignoseric acid (C24:0)	0.22 ± 0.01 ^c^	0.15 ± 0.01 ^a^	0.17 ± 0.01 ^ab^	0.18 ± 0.02 ^b^
	Saturated fatty acids (SFAs)	27.37	27.13	26.02	26.26
	Monounsaturated fatty acids (MUFAs)	61.26	61.62	61.54	61.33
	Polyunsaturated fatty acids (PUFAs)	11.36	11.25	12.44	12.41
	Unsaturated fatty acids (MUFAs + PUFAs)	72.62	72.87	73.98	73.74

* Results are the mean value of three replications and standard deviation (mean ± std dev.) of fatty acid. Concentration of oil and microcapsule samples with different lowercase letters (a–c) in the same row are statistically significant (*p* < 0.05).

**Table 7:** Aroma compounds of NY olive oil and microcapsule products during storage (0, 45, and 90 days) (µg/kg).

No.	Aroma Compounds	RT	NY			NY1:1			NY1:4			NY1:10		
			0 Days	45 Days	90 Days	0 Days	45 Days	90 Days	0 Days	45 Days	90 Days	0 Days	45 Days	90 Days
1	Hexanal	13.35	5711 ± 51 ^aC^	6046 ± 72 ^aD^	6646 ± 176 ^bB^	1481 ± 15 ^bA^	1234 ± 12 ^aA^	1227 ± 14 ^aA^	1463 ± 18 ^cA^	1366 ± 13 ^bB^	1283 ± 19 ^aA^	2812 ± 20 ^cB^	1976 ± 23 ^bC^	1471 ± 19 ^aA^
2	Heptanal	18.39	2620 ± 116 ^a^	3682 ± 99 ^b^	3741 ± 101 b	nd	nd	nd	nd	nd	nd	nd	nd	nd
3	*D*-Limonene	19.44	3855 ± 33 ^aC^	3886 ± 58 ^aB^	3921 ± 178 ^aA^	3214 ± 54 ^aA^	3549 ± 105 ^abAB^	3653 ± 182 ^bA^	3459 ± 160 ^aAB^	3478 ± 169 ^aA^	3611 ± 133 ^aA^	3730 ± 173 ^aBC^	3527 ± 174 ^aAB^	3530 ± 168 ^aA^
4	(*E*)-2-Hexenal	19.82	366 ± 18 ^aB^	864 ± 17 ^bB^	917 ± 27 ^bC^	81 ± 9 ^aA^	74 ± 9 ^aA^	62 ± 5 ^aA^	86 ± 6 ^aA^	80 ± 5 ^aA^	71 ± 4 ^aA^	96 ± 7 ^bA^	85 ± 6 ^abA^	74 ± 4 ^aA^
5	2-Pentyl furan	20.83	175 ± 6 ^a^	408 ± 18 ^b^	479 ± 23 c	nd	nd	nd	nd	nd	nd	nd	nd	nd
6	Pentanol	21.48	700 ± 20 ^a^	744 ± 18 ^a^	907 ± 20 c	nd	nd	nd	nd	nd	nd	nd	nd	nd
7	*Ɣ*-Terpinene	21.76	nd	nd	nd	1986 ± 40 ^aA^	3579 ± 171 ^bA^	4912 ± 105 ^cA^	1866 ± 49 ^aA^	4383 ± 114 ^bB^	5215 ± 124 ^cAB^	2949 ± 65 ^aB^	5015 ± 171 ^bC^	5297 ± 151 ^bC^
8	3,7-Dimethyl octanol	22.19	1382 ± 26 ^a^	1512 ± 32 ^ab^	1614 ±58 ^b^	nd	nd	nd	nd	nd	nd	nd	nd	nd
9	*p*-Cymene	22.77	nd	nd	nd	1764 ± 45 ^aA^	2317 ± 46 ^bA^	2730 ± 83 ^cA^	1815 ± 30 ^aA^	2878 ± 119 ^bB^	3085 ± 41 ^bB^	2889 ± 73 ^aB^	2940 ± 92 ^abB^	3270 ± 136 ^bB^
10	Hexyl acetate	22.86	185 ± 9 ^a^	212 ± 14 ^a^	316 ± 16 ^b^	nd	nd	nd	nd	nd	nd	nd	nd	nd
11	Octanal	23.60	3684 ± 32 ^aB^	6352 ± 80 ^bB^	7367 ± 92 ^cB^	146 ± 8 ^aA^	244 ± 19 ^bA^	288 ± 20 ^bA^	nd	nd	nd	nd	nd	nd
12	(*E*)-2-Heptenal	25.01	517 ± 18 ^a^	1205 ± 27 ^b^	1692 ± 96 ^c^	nd	nd	nd	nd	nd	nd	nd	nd	nd
13	6-Methyl-5-Hepten-2-one	25.55	nd	218 ± 10 ^a^	379 ± 11 ^b^	nd	nd	nd	nd	nd	nd	nd	nd	nd
14	Hexanol	26.26	5266 ± 88 ^aD^	6000 ± 76 ^bD^	6265 ± 55 ^cD^	1565 ± 56 ^bA^	1383 ± 92 ^abA^	1276 ± 20 ^aA^	2086 ± 76 ^bB^	1917 ± 31 ^aB^	1871 ± 35 ^aB^	2644 ± 88 ^aC^	2576 ± 150 ^aC^	2496 ± 125 ^aC^
15	(*Z*)-3-Hexenol	27.47	167 ± 10 ^a^	190 ± 13 ^a^	nd	nd	nd	nd	nd	nd	nd	nd	nd	nd
16	Nonanal	28.35	2432 ± 73 ^aA^	4101 ± 192 ^bC^	5859 ± 51 ^cC^	366 ± 12 ^aB^	481 ± 22 ^bA^	529 ± 25 ^bA^	409 ± 22 ^aB^	685 ± 17 b^AB^	612 ± 33 b^AB^	632 ± 32 ^aC^	870 ± 21 ^bB^	653 ± 35 ^aB^
17	(*E*)-2-Octenal	29.62	251 ± 19 ^a^	661 ± 10 ^b^	1137 ± 42 ^c^	nd	nd	nd	nd	nd	nd	nd	nd	nd
18	2-Butyl octanol	29.70	nd	nd	nd	233 ± 10 ^aA^	350 ± 23 ^bAB^	614 ± 38 ^cC^	213 ± 15 ^aA^	321 ± 19 ^bA^	436 ± 20 ^cA^	224 ± 14 ^aA^	402 ± 26 ^bB^	492 ± 24 ^cA^
19	Acetic acid	29.74	1921 ± 34 ^a^	3132 ± 45 ^b^	1791 ± 51 ^a^	nd	nd	nd	nd	nd	nd	nd	nd	nd
20	Heptanol	30.50	1312 ± 23 a	1868 ± 77 b	2221 ± 131 c	nd	nd	nd	nd	nd	nd	nd	nd	nd
21	(*E, E*)-2,4-Heptadienal	30.73	332 ± 2 ^a^	668 ± 11 ^b^	1116 ± 45 ^c^	nd	nd	nd	nd	nd	nd	nd	nd	nd
22	2-Octynoic acid	31.89	nd	145 ± 8 ^a^	346 ± 10 ^b^	nd	nd	nd	nd	nd	nd	nd	nd	nd
23	Decanal	32.61	nd	nd	343 ± 18	nd	nd	nd	nd	nd	nd	nd	nd	nd
24	(*E*)-2-Heptenol	32.64	137 ± 9 ^a^	250 ± 19 ^b^	302 ± 19 ^b^	nd	nd	nd	nd	nd	nd	nd	nd	nd
25	Benzaldehyde	33.03	nd	nd	nd	314 ± 13 ^cA^	256 ± 17 ^aA^	288 ± 21 ^abA^	289 ± 20 ^aA^	367 ± 23 ^bA^	368 ± 18 ^bB^	2262 ± 65 ^bB^	2689 ± 91 ^cB^	1464 ± 45 ^aC^
26	Copaene	33.24	208 ± 14 ^a^	455 ± 14 ^b^	711 ± 38 ^c^	nd	nd	nd	nd	nd	nd	nd	nd	nd
27	(*E*)-2-Nonenal	34.12	nd	144 ± 9 ^a^	266 ± 14 ^b^	nd	nd	nd	nd	nd	nd	nd	nd	nd
28	Octanol	34.98	705 ± 25 ^a^	1089 ± 21 ^b^	1651 ± 72 ^c^	nd	nd	nd	nd	nd	nd	nd	nd	nd
29	(*E*)-2-Decenal	38.95	482 ± 18 ^a^	997 ± 21 ^b^	1639 ± 30 ^c^	nd	nd	nd	nd	nd	nd	nd	nd	nd
30	Hexanoic acid	45.23	1212 ± 35 ^a^	1775 ± 103 ^b^	2458 ± 66 ^c^	nd	nd	nd	nd	nd	nd	nd	nd	nd
31	Phenylethyl alcohol	47.13	164 ± 5 ^a^	386 ± 15 ^b^	450 ± 26 ^c^	nd	nd	nd	nd	nd	nd	nd	nd	nd
32	Octanoic acid	51.08	nd	nd	609 ± 20	nd	nd	nd	nd	nd	nd	nd	nd	nd
33	Nonanoic acid	53.63	nd	nd	809 ± 24	nd	nd	nd	nd	nd	nd	nd	nd	nd

Lowercase letters (a, b, c, and d) indicate statistical differences between storage times (0, 45, and 90) within the same sample group. Uppercase letters (A, B, C, and D) indicate statistical differences observed between different groups at the same storage time. nd: not determined.

**Table 8:** Aroma compounds of KY olive oil and microcapsule products during storage (0, 45, and 90 days) (µg/kg).

No.	Aroma Compounds	RT	KY			KY1:1			KY1:4			KY1:10		
			0 Days	45 Days	90 Days	0 Days	45 Days	90 Days	0 Days	45 Days	90 Days	0 Days	45 Days	90 Days
1	Hexanal	13.35	3582 ± 23 ^bB^	3542 ± 18 ^abB^	3503 ± 24 ^aC^	1514 ± 22 ^cA^	1253 ± 13 ^bA^	1154 ± 11 ^aA^	1503 ± 22 ^cA^	1334 ± 19 ^bA^	1201 ± 14 ^aB^	1495 ± 26 ^cA^	1427 ± 19 ^bA^	1233 ± 14 ^aB^
2	2-Pentanol	15.15	166 ± 13 ^bA^	115 ± 7 ^aA^	136 ± 10 ^abA^	nd	nd	nd	nd	nd	nd	nd	nd	nd
3	Ethyl pentanoate	16.02	215 ± 10 ^b^	100 ± 6 ^a^	116 ± 9 ^a^	nd	nd	nd	nd	nd	nd	nd	nd	nd
4	2-Heptanone	18.18	nd	nd	132 ± 9 ^aA^	nd	nd	nd	nd	nd	nd	nd	nd	nd
5	Heptanal	18.39	412 ± 19 ^bA^	303 ± 9 ^aA^	502 ± 17 ^cA^	nd	nd	nd	nd	nd	nd	nd	nd	nd
6	Methyl hexanoate	18.53	834 ± 19 ^bA^	580 ± 13 ^aA^	542 ± 15 ^aA^	nd	nd	nd	nd	nd	nd	nd	nd	nd
7	*D*-Limonene	19.44	3315 ± 24 ^aA^	3589 ± 24 ^bA^	3596 ± 110 ^cC^	2125 ± 142 ^aB^	2655 ± 100 ^bC^	2771 ± 106 ^bB^	2324 ± 140 ^aBC^	2385 ± 103 ^aBC^	2672 ± 122 ^aA^	2326 ± 107 ^aC^	2476 ± 90 ^aB^	2557 ± 140 ^aA^
8	(*E*)-2-Hexenal	19.82	546 ± 19 ^aB^	700 ± 18 ^b^	849 ± 21 ^c^	320 ± 18 ^aA^	nd	nd	nd	nd	nd	nd	nd	nd
9	2-Pentyl furan	20.83	96 ± 7 ^a^	105 ± 8 ^a^	152 ± 7 ^b^	nd	nd	nd	nd	nd	nd	nd	nd	nd
10	Ethyl hexanoate	21.01	500 ± 17 ^bA^	411 ± 15 ^a^	554 ± 22 ^b^	nd	nd	nd	nd	nd	nd	nd	nd	nd
11	Pentanol	21.48	278 ± 13 ^b^	150 ± 11 ^a^	279 ± 17 ^b^	nd	nd	nd	nd	nd	nd	nd	nd	nd
12	*Ɣ*-Terpinene	21.76	nd	nd	nd	1734 ± 30 ^aA^	3842 ± 87 ^bA^	5252 ± 89 ^cA^	2435 ± 77 ^aB^	4899 ± 162 ^bC^	5771 ± 287 ^cB^	1899 ± 95 ^aA^	4361 ± 70 ^bB^	5098 ± 176 ^cA^
13	Linalyl isobutyrate	21.97	115 ± 8 ^a^	117 ± 7 ^a^	241 ± 13 ^b^	nd	nd	nd	nd	nd	nd	nd	nd	nd
14	*p*-Cymene	22.77	nd	nd	nd	1581 ± 53 ^aA^	2151 ± 48 ^bA^	2880 ± 93 ^cA^	2310 ± 76 ^aA^	2561 ± 89 ^aB^	3037 ± 121 ^bA^	1978 ± 91 ^aC^	2762 ± 84 ^bC^	3078 ± 67 ^cA^
15	Hexyl acetate	22.86	432 ± 18 ^a^	484 ± 16 ^a^	649 ± 22 ^b^	nd	nd	nd	nd	nd	nd	nd	nd	nd
16	Octanal	23.60	1687 ± 21 ^aC^	1966 ± 75 ^bC^	2805 ± 36 ^cD^	172 ± 11 ^aA^	286 ± 19 ^bA^	377 ± 11 ^cA^	187 ± 11 ^aA^	375 ± 16 ^bA^	570 ± 21 ^cB^	448 ± 22 ^aB^	579 ± 25 ^bB^	688 ± 33 ^cC^
17	(*Z*)-2-Pentenol	24.60	134 ± 9 ^a^	157 ± 10 ^a^	226 ± 11 ^b^	nd	nd	nd	nd	nd	nd	nd	nd	nd
18	2-Nonynoic acid	24.73	247 ± 10 ^a^	291 ± 19 ^a^	398 ± 20 ^b^	nd	nd	nd	nd	nd	nd	nd	nd	nd
19	6-Methyl-5-Hepten-2-one	25.41	nd	189 ± 14 ^a^	264 ± 16 ^b^	nd	nd	nd	nd	nd	nd	nd	nd	nd
20	Hexanol	26.26	5536 ± 84 ^cD^	3409 ± 61 ^aD^	4976 ± 60 ^bD^	655 ± 25 ^bB^	613 ± 18 ^abB^	574 ± 21 ^aB^	421 ± 24 ^cA^	397 ± 21 ^bA^	373 ± 12 ^aA^	845 ± 31 ^aC^	816 ± 34 ^aC^	754 ± 20 ^aC^
21	(*Z*)-3-Hexenol	27.47	2230 ± 44 ^b^	1478 ± 26 ^a^	2237 ± 52 ^b^	nd	nd	nd	nd	nd	nd	nd	nd	nd
22	Methyl octanoate	28.16	308 ± 17 ^a^	347 ± 9 ^a^	460 ± 20 ^b^	nd	nd	nd	nd	nd	nd	nd	nd	nd
23	Nonanal	28.35	2312 ± 67 ^aC^	2488 ± 78 ^aC^	3029 ± 52 ^bD^	398 ± 28 ^aA^	634 ± 27 ^bA^	655 ± 13 ^bA^	440 ± 28 ^aA^	719 ± 26 ^bA^	967 ± 36 ^cC^	1262 ± 58 ^cB^	964 ± 30 ^bB^	810 ± 23 ^aB^
24	(*E*)-2-Octenal	29.62	94 ± 8 ^a^	107 ± 5 ^a^	156 ± 8 ^b^	nd	nd	nd	nd	nd	nd	nd	nd	nd
25	Acetic acid	29.74	3292 ± 66 ^c^	2444 ± 65 ^b^	1348 ± 38 ^a^	nd	nd	nd	nd	nd	nd	nd	nd	nd
26	Heptanol	30.50	1476 ± 16 ^b^	1248 ± 73 ^a^	1891 ± 59 ^c^	nd	nd	nd	nd	nd	nd	nd	nd	nd
27	Decanal	32.61	nd	118 ± 6 ^a^	112 ± 7 ^a^	nd	nd	nd	nd	nd	nd	nd	nd	nd
28	Benzaldehyde	33.03	nd	nd	nd	1644 ± 45 ^aB^	2340 ± 43 ^aC^	1707 ± 44 ^bB^	375 ± 24 ^bA^	421 ± 31 ^bA^	189 ± 12 ^aB^	2008 ± 45 ^bC^	2135 ± 43 ^bA^	1656 ± 88 ^aB^
29	Copaene	33.24	572 ± 20 ^a^	563 ± 17 ^a^	853 ± 20 ^b^	nd	nd	nd	nd	nd	nd	nd	nd	nd
30	Octanol	34.98	841 ± 29 ^a^	797 ± 21 ^a^	1267 ± 49 ^b^	nd	nd	nd	nd	nd	nd	nd	nd	nd
31	(*E*)-2-Decenal	38.95	542 ± 22 ^a^	758 ± 9 ^b^	511 ± 19 ^a^	nd	nd	nd	nd	nd	nd	nd	nd	nd
32	Hexanoic acid	45.23	494 ± 27 ^b^	445 ± 20 ^b^	235 ± 18 ^a^	nd	nd	nd	nd	nd	nd	nd	nd	nd
33	Phenylethyl alcohol	47.13	138 ± 5 ^a^	260 ± 9 ^b^	224 ± 16 ^c^	nd	nd	nd	nd	nd	nd	nd	nd	nd
34	Octanoic acid	51.08	nd	nd	106 ± 8 ^a^	nd	nd	nd	nd	nd	nd	nd	nd	nd
35	Nonanoic acid	53.63	nd	nd	87 ± 4 ^a^	nd	nd	nd	nd	nd	nd	nd	nd	nd

Lowercase letters (a, b, c, and d) indicate statistical differences between storage times (0, 45, and 90) within the same sample group. Uppercase letters (A, B, C, and D) indicate statistical differences observed between different groups at the same storage time. nd: not determined.

## Data Availability

The original contributions presented in the study are included in the article, further inquiries can be directed to the corresponding authors.
